# Coadministration of Resveratrol and Rice Oil Mitigates Nociception and Oxidative State in a Mouse Fibromyalgia-Like Model

**DOI:** 10.1155/2016/3191638

**Published:** 2016-03-16

**Authors:** Caroline Peres Klein, Marcos Rodrigues Cintra, Nancy Binda, Danuza Montijo Diniz, Marcus Vinicius Gomez, Andre Arigony Souto, Alessandra Hubner de Souza

**Affiliations:** ^1^Postgraduate Program in Cellular and Molecular Biology, Pontifical Catholic University of Rio Grande do Sul, 90619-900 Porto Alegre, RS, Brazil; ^2^Postgraduate Program in Genetics and Applied Toxicology, Lutheran University of Brazil, 92425-900 Canoas, RS, Brazil; ^3^Postgraduate Program in Health Sciences: Medicine and Biomedicine, Institute of Education and Research, Grupo Santa Casa de Belo Horizonte, 30150-240 Belo Horizonte, MG, Brazil; ^4^Faculty of Chemistry, Pontifical Catholic University of Rio Grande do Sul, 90619-900 Porto Alegre, RS, Brazil; ^5^Postgraduate Program in Cellular and Molecular Biology Applied to Health, Lutheran University of Brazil, 92425-900 Canoas, RS, Brazil

## Abstract

The mechanism underlying pain symptoms in fibromyalgia (FM) is not fully understood. Oxidative stress has emerged as pathophysiological event occurring during the development of the disease. The present study aimed at investigating the efficacy of resveratrol associated with rice bran oil on fibromyalgia-like mice model. Subcutaneous injection of reserpine (0.25 mg/Kg) during 3 days produced fibromyalgia-like symptoms. Resveratrol and/or rice oil or pregabalin were administered through oral route in therapeutic (single dose) and preventive (four doses) schemes. In both schemes, treatment with resveratrol associated with rice bran oil and pregabalin significantly reduced mechanical allodynia and thermal hyperalgesia in animals. The preventive scheme displayed antidepressant effect which was demonstrated by the forced swimming test as well as reduced reactive species in the cerebrospinal fluid of reserpinized animals. Taken together, our data provide evidences that the intake of resveratrol associated with rice bran oil plays antinociceptive and antidepressant actions probably through reducing reactive species and suggests the involvement of oxidative stress in this model of FM as possible underlying mechanism of pathogenesis of the disease.

## 1. Introduction

Painful syndromes are highly prevalent among populations around the world. Fibromyalgia (FM) is a common disorder characterized by chronic widespread pain along the body, especially prevalent in women [1, 2].Beyond the pain as cardinal symptom in specific anatomical sites, FM patients complain of other debilitating conditions such as fatigue, anxiety, depression, and sleep disturbances [3, 4]. Additionally, FM can be present with other comorbidities [5, 6], including irritable bowel syndrome [7], interstitial cystitis [8], chronic fatigue syndrome [9], and temporomandibular syndrome [10]. The heterogeneity of FM makes it difficult to understand its pathophysiology. To date, the available therapeutic approaches for FM have limited effects once the side effects can be allied to the treatment [11] or even the treatment should be individualized [12].

Nagakura et al. [13] validated a fibromyalgia-like model in rats induced by repeated subcutaneous injection of reserpine (RES) and, recently, we have published data of an adapted model for mice [14, 15]. Notwithstanding, a fibromyalgia-like model in animal is difficult, given the multiple causes and syndromes with symptoms similar to those of FM [16]. The present fibromyalgia-like model mimics fibromyalgia-related symptoms as nociception and depression. In addition, the drugs used in the clinic to treat FM have shown effects in reversing the painful symptoms in the reserpine-injected animals [13].

Growing evidences have shown the antioxidant compounds have antinociceptive effects in several clinical trials [17] and in animal models with neuropathic [18] and inflammatory pain [19]. In addition to their antinociceptive effects, antioxidant compounds also show antifatigue and anxiolytic ability, both present in fibromyalgic patients, as demonstrated by both human [20] and animal studies [21, 22].

Resveratrol (RSV) is a polyphenol produced as a defensive molecule against stress and injury in plants [23] and also displays versatile pharmacological effects [24]. RSV has been employed as a commercial nutraceutical product, once it is derived from a wide range of plants, especially grapes [25], and it is present in red wine [26]. Remarkably, RSV exerts antioxidant [27], anti-inflammatory [28], antinociceptive [29], neuroprotective [30], chemopreventive [31], hepatoprotective [29], and cardioprotective activities [32] through minimizing reactive species levels and improving antioxidant properties of the system. By the other side, rice bran oil (RO) is also a natural product; it is an enriched source of vitamin E (tocopherols and tocotrienols) and derivatives esterified (oryzanol) [33]. Few data are available concerning RO abilities. However, it has been demonstrated that RO presents antioxidant [34], immunomodulatory [33], anti-inflammatory [35], hypolipidemic [36], and anticancer properties [37]. Recently, a formulation containing RSV and RO was proved to act synergistically through increasing therapeutic potential of both compounds, displaying antioxidant, anti-inflammatory, chemopreventive, and neuroprotective effects [38].

To the best of our knowledge, there is not any evidence in the literature showing the effects of RSV in FM, in neither clinical nor animal studies. Therefore, the present work aimed to investigate whether the RSV and/or the association between RSV and RO in a single formulation might modulate behavioral and biochemical changes in a fibromyalgia-like mouse model induced by reserpine following an acute or repeated administration dosed by oral route.

Our results point to the hypothesis of the involvement of oxidative stress in the pathogenesis of FM and the association of RSV and RO as a potential approachas nutraceutical products to treating FM symptoms.

## 2. Material and Methods

### 2.1. Drugs

The following drugs were used to perform the present work: pregabalin (Lyrica, Pfizer, UK), resveratrol and rice oil (Faculty of Chemistry, PUCRS), all solubilized in sterile saline solution (NaCl 0.9%), and reserpine (Sigma Chemical Company, St. Louis, USA), which was solubilized in acetic acid 0.05% and distillated water (V/V).

### 2.2. Animals

In this study, male Swiss mice weighing 30 to 35 g were used, maintained on a 12 h light/dark cycle (light on at 7:00 am) at 22 ± 2°C under controlled humidity (60 to 70%) with food and water provided* ad libitum*. In all experiments, the animals were acclimatized to the laboratory for at least 1 h before testing. Experiments were conducted in accordance with the National Institute for Health (NIH) guidelines. All efforts were made to minimize animal suffering and to keep the number of animals to a minimum for demonstrating consistent effects for the treatments. The experimental protocols were approved by the local animal committee under number 2012-18P (CEUA-ULBRA).

### 2.3. Induction of Fibromyalgia (FM)

Fibromyalgia-like model was induced according to the method described by Nagakura et al. [13] for rats, which was adapted for mice [14]. The administration of reserpine (0.25 mg/kg), given by subcutaneous route (s.c.), once a day, during three consecutive days caused the amine depletion. Control groups received vehicle (saline solution), employing the same schedule of administration. Following reserpine administration, on the 4th day the animals were subjected to the behavioral tests.

### 2.4. Experimental Groups

We aimed to investigate the effect of resveratrol plus rice oil in comparison to isolated treatment using resveratrol or rice oil dosed in single or repeated doses on behavioral changes elicited by reserpine. For both, RSV and RO, research has demonstrated health-promoting properties of each compound, specifically their antinociceptive and antioxidative actions [27, 34]. Combining RSV with RO was shown to increase the therapeutic effect of RSV in at least an additive manner, and the delayed dispersion of RSV to the system in the blended formulation likely contributed to the lack of observed side effects [38]. Pregabalin (PGB) was used as a positive drug control and saline solution (SAL; NaCl 0.9%, 10 mL/kg, p.o.) was used as negative control.

In the first set of experiments all treatments were given in a therapeutic scheme (single dose) through gavage, 60 min before the experimental assessments, in the following doses: SAL (10 mL/kg); RSV (100 mg/kg, p.o.) [38]; RO (10 mL/kg, p.o.) [38]; RSV plus RO (10 mg/kg +10 mL/kg, p.o.) [38]; or PGB (30 mg/kg, p.o.) [22].

In the second experimental set, we evaluated the effects of repeated administration (preventive scheme) of SAL, RSV, RO, RSV + RO, and PGB, in the same doses described above, during three consecutive days, 30 min after a daily reserpine injection. On the 4th day, mice also received the respective drug treatment, dosed 60 min before behavioral evaluation.

Behavioral assessment was performed blindly with respect to drug administration. The animals were used for one procedure only. On completion of testing mice were euthanized by cervical dislocation, with exception for RS measurement, where they were euthanized with isoflurane for collection of 20 *μ*L of cerebrospinal fluid (CSF) through cisterna magna puncture [39].

### 2.5. Behavioral Tests

#### 2.5.1. Mechanical Allodynia

Mechanical allodynia thresholds were measured using Von Frey filaments applied to the hind paw plantar surface through the up-down paradigm originally described by Dixon [40] and the method described by de Souza et al. [14] for mice. Mice were acclimatized for 60 min prior to the test. The paw withdrawal threshold was expressed in grams (g) and was evaluated before (basal records) and on the 4th day after induction of FM. A significant decrease in paw withdrawals threshold compared to baseline values was considered as mechanical allodynia.

#### 2.5.2. Hot Plate Test

The hot plate test was used to measure the antinociceptive effects of drugs following the methodology described by Hunskaar et al. [41], with slight modifications. The surface of the hot plate was heated to a constant temperature of 50 ± 0.1°C. Following the appropriate treatments, mice were placed on the hot plate apparatus (Ugo Basile, Italy). The latency (s) to respond with hind paw licking, hind paw flick, or jump (whichever came first) was measured and indicated nociceptive behavior in response to thermal stimulus. Trials were terminated if the animals did not respond within 30 s, to prevent tissue damage.

#### 2.5.3. Open Field Test

The experiments in the open field were conducted as originally described by Holland and Weldon [42]. On the 4th day after the onset of treatments, mice were individually placed in the center of an acrylic box (40 × 60 × 50 cm), with the floor divided into 12 squares, in a sound-attenuated room, under low intensity light. The number of squares crossed with the four paws was recorded, during a period of 5 min.

#### 2.5.4. Forced Swimming Test

We used the same methodology previously described by Porsolt et al. [43]. The experiments were carried out using a polyvinyl chloride (PVC) cylinder (18.5 cm diameter, 25 cm height) filled with water to the height of 17 cm. Water was maintained at 23 ± 2°C. Mice were placed into the water to quantify the immobility time, which was defined as an absence of all movements except motions required for keeping the mouse's head above the water. The time during which mice remained immobile was recorded, in seconds, during a period of 2 min.

### 2.6. Biochemical Assays

#### 2.6.1. Serum Transaminases Levels

To assess liver function, serum alanine aminotransferase (ALT) and aspartate aminotransferase (AST) levels were determined using commercial kits (Labtest, Lagoa Santa, Brazil).

#### 2.6.2. Reactive Species (RS) Levels in the Cerebrospinal Fluid (CSF)

RS level was determined through the dichlorofluorescein (DCFH) oxidation, formation, according to the method described by LeBel et al. [44]. Serum was incubated with 2′,7′-dichlorofluorescein diacetate (H2DCF-DA). H2DCF-DA is a cleaved and dissociated to the product H2DCF; H2DCF is oxidized by reactive species present in the sample, producing fluorescent DCF. In the function of the time, DCF was determined in a wavelength of 488 nm for excitation and 525 nm for emission. Data are presented as arbitrary unit (AU).

### 2.7. Statistical Analysis

Data were analyzed and plotted on graphs using the GraphPad Prism 6.0 software (San Diego, CA, USA). Data were analyzed by one-way analysis of variance (ANOVA) followed by Bonferroni* post hoc* test and expressed as mean ± standard error mean. Values with *p* < 0.05 were considered significant.

## 3. Results

### 3.1. Antiallodynic Effect of Single Administration of RSV, RO-Combined RSV, or PGB on a Fibromyalgia Model in Mice

**Figure 1:**
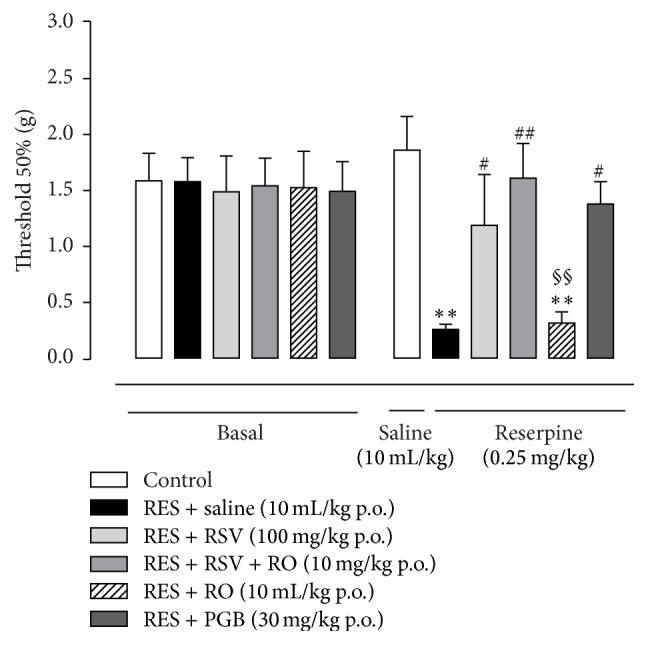
Antiallodynic effects of acute treatment with RSV, RSV + RO, and PGB, dosed orally, on hindpaw withdrawal threshold (g) to mechanical stimulation in the fibromyalgia model induced by reserpine in mice. Pregabalin was used as a positive control drug. Mechanical hypersensitivity was assessed before (baseline) and on the 4th day after onset of reserpine administration (0.25 mg/kg, s.c., once a day for three days). Data are presented as mean ± SEM. ^*∗∗*^ *p* < 0.001 compared to control (SAL-SAL), ^#^ *p* < 0.05 compared to RES-SAL group, ^##^ *p* < 0.01 compared to RES-SAL group, and ^§§^ *p* < 0.01 compared to RES-RSV-RO. Statistical analysis was performed by one-way ANOVA followed by Bonferroni's* post hoc* test. *n* = 7 to 11 mice/group.

On the 4th day after the reserpine injections (0.25 mg/kg for three consecutive days), mice presented a reduction in the mechanical threshold tested by Von Frey filaments in comparison to the control group (*p* < 0.001, Figure 1), indicating mechanical allodynia. RSV isolated (100 mg/kg) or PGB (30 mg/kg) treatment increased the mechanical threshold when compared to the RES-SAL group (*p* < 0.05). RO-combined RSV (10 mg/kg) treatment induced increasing in the mechanical threshold above the control group (*p* < 0.01, Figure 1). Conversely, RO treatment alone was ineffective in altering the mechanical threshold when compared to the RES-SAL group (*p* > 0.05).

### 3.2. Antihyperalgesic Effect of Single Administration of RO-Combined RSV or PGB on a Fibromyalgia Model in Mice

**Figure 2:**
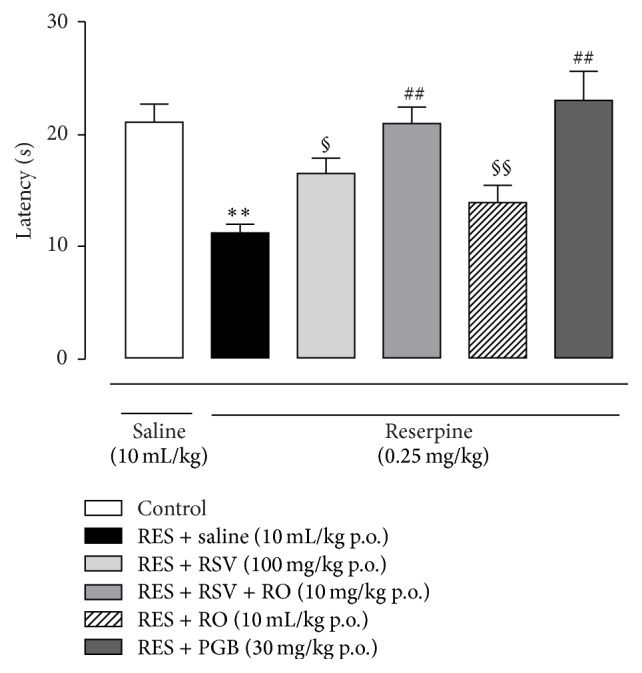
Antinociceptive effects of acute treatment with RSV + RO or PGB on latency time (s) in response to thermal stimulation in the fibromyalgia model. PGB was used as a positive control drug. Thermal hyperalgesia was assessed in the hot plate test, on the 4th day after onset of reserpine administration (0.25 mg/kg, s.c., once a day for three days), 30 min after treatment administration. Data are presented as mean ± SEM. ^*∗∗*^ *p* < 0.001 compared to control (SAL-SAL), ^##^ *p* < 0.01 compared do RES-SAL group, ^§^ *p* < 0.05 compared to RES-RSV-RO, and ^§§^ *p* < 0.01 compared to RES-RSV-RO. Statistical analysis was performed by one-way ANOVA followed by Bonferroni's* post hoc* test. *n* = 7 to 11 mice/group.

Similarly to mechanical allodynia, on the 4th day s.c. reserpine injections (0.25 mg/kg for three consecutive days) produced a reduction in paw withdrawal latency (s) in the hot plate test of mice compared to the control group (*p* < 0.001, Figure 2), indicating thermal hyperalgesia. Treatments with RO-combined RSV (10 mg/kg) or PGB (30 mg/kg) increased paw withdrawal latencies to values comparable to the control group, reversing the thermal hyperalgesia produced by reserpine. The combination of RO and RSV (10 mg/kg) also increased latencies significantly compared to the treatment with RSV or RO only (*p* < 0.05 and *p* < 0.01, resp., Figure 2). Concerning RSV (100 mg/kg), although the treatment reduced the latency time, it was not able to reverse the effect of reserpine (*p* > 0.05). The RO (10 mL/kg) treatment also did not alter the effect of reserpine.

### 3.3. Single Dose Administration of RSV, RO-Combined RSV, or PGB Has No Effect on the Open Field Test in the Fibromyalgia Model Induced by Reserpine in Mice

**Figure 3:**
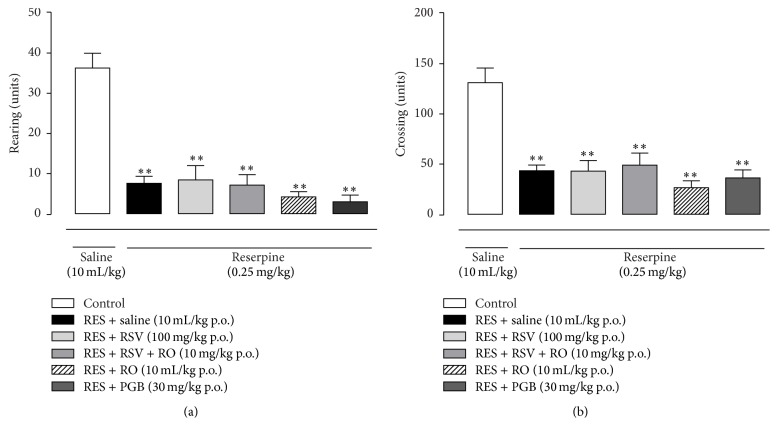
Effect of RSV, RSV + RO, or PGB on the locomotor activity in the fibromyalgia model induced by reserpine in mice. PGB was used as a positive control drug. Spontaneous locomotor activity was assessed in the open field test on the 4th day after onset of reserpine administration (0.25 mg/kg, s.c., once a day for three days). Data are presented as mean ± SEM. ^*∗∗*^ *p* < 0.001 compared to control (SAL-SAL). Statistical analysis was performed by one-way ANOVA followed by Bonferroni's* post hoc* test. *n* = 7 to 11 mice/group.

Locomotive activity parameters of mice, as crossing and rearing counts, were significantly diminished on the 4th day after the s.c. reserpine injections (0.25 mg/kg for three consecutive days) compared to the control group (*p* < 0.001, Figures 3(a) and 3(b)). None of these parameters were reversed after the treatment with RSV (100 mg/kg), RSV + RO (10 mg/kg), RO (10 mL/kg), or PGB (30 mg/kg), and no difference was achieved when compared to the RES-SAL group (*p* > 0.05, Figures 3(a) and 3(b)).

### 3.4. Repeated Administration of RO-Combined RSV or PGB Prevents Mechanical Allodynia and Thermal Hyperalgesia in the Fibromyalgia Model in Mice

**Figure 4:**
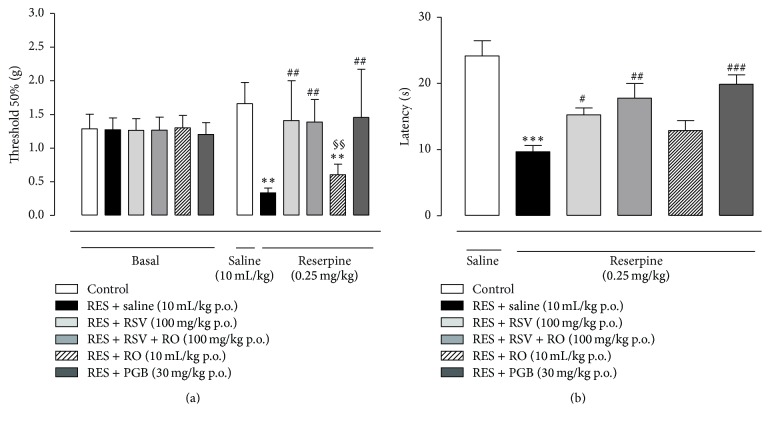
Effects of chronic treatment with RSV, RSV + RO, or PGB on reserpine-induced nociception in the fibromyalgia model in mice. On the 4th day, mice receiving reserpine (0.25 mg/kg, s.c., once a day for 3 days) and treated orally with RSV (100 mg/kg once a day for 4 days), RSV + RO (10 mg/kg once a day for 4 days), RO (10 mL/kg once a day for 4 days), or PGB (30 mg/kg once a day for 4 days) were tested through Von Frey stimulation for mechanical allodynia (a) and in the open field test for thermal hyperalgesia (b). Data are presented as mean ± SEM. ^*∗∗∗*^ *p* < 0.001 compared to control (SAL-SAL), ^#^ *p* < 0.05, ^##^ *p* < 0.01, and ^###^ *p* < 0.001 compared to RES-SAL group, and ^§§^ *p* < 0.01 compared to RES-RSV-RO. Statistical analysis was performed by one-way ANOVA followed by Bonferroni's* post hoc* test. (a) *n* = 9 to 13 mice/group; (b) *n* = 7 to 8 mice/group. ^*∗∗*^ *p* < 0.01 compared to control (SAL-SAL).

In another set of experiments, we investigated the effects of chronic treatment with RSV (100 mg/kg once a day for four days), RSV + RO (10 mg/kg once a day for four days), or PGB (30 mg/kg once a day for four days) on reserpine-related nociception. On the 4th day we observed that the chronic treatment with RSV, RSV + RO, or PGB increased the threshold to 50% in the animals when compared to mice receiving saline only (RES-SAL group), presenting an antiallodynic effect (*p* < 0.01, Figure 4(a)). In addition, the chronic treatment with RSV (*p* < 0.05, Figure 4(b)), RSV + RO or PGB (*p* < 0.01, Figure 4(b)) increased the latency (s) of paw withdrawal in the hot plate test, preventing the reserpine-induced thermal nociception. Chronic administration of RO only (10 mL/kg once a day for four days) did not inhibit mechanical nociception induced by reserpine but did demonstrate a tendency to inhibit thermal nociception in comparison to RES-SAL group (*p* = 0.0508, Figure 4(b)). Chronic treatment with RSV + RO showed similar trends as the RSV group in affecting mechanical and thermal nociception (Figure 4).

### 3.5. Evaluation of Repeated Administration of RSV, RO-Combined RSV, or PGB in the Open Field Test in the Fibromyalgia Model in Mice

**Figure 5:**
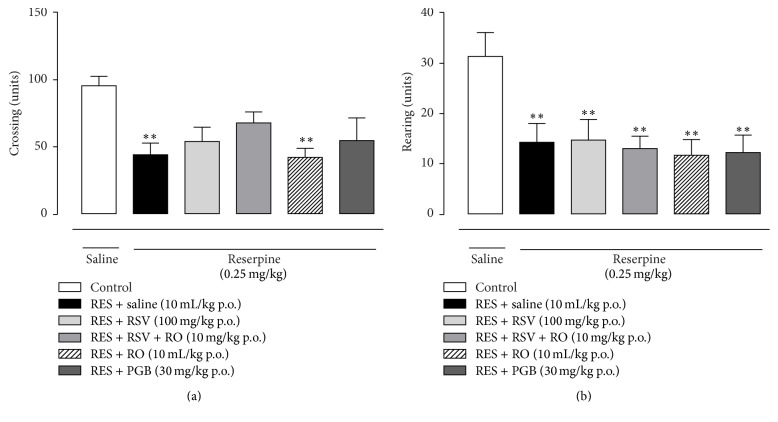
Assessment of chronic treatment with RSV, RSV + RO, or PGB on the open field test in the fibromyalgia model in mice. The exploratory ability of the mice, crossing (a) and rearing (b), was tested on the 4th day after onset of reserpine injection (0.25 mg/kg, s.c., once a day for 3 days) and after the onset of the oral administration of its corresponding treatment (once a day for 4 days). Data are presented as mean ± SEM. ^*∗∗*^ *p* < 0.01 compared to control (SAL-SAL). Statistical analysis was performed by one-way ANOVA followed by Bonferroni's* post hoc* test. (a and b) *n* = 6 to 10 mice/group.

Some of the tested treatments administered chronically during four consecutive days (RSV, RSV + RO, or PGB) also partially interfered with the reserpine-induced diminishment of crossing numbers in the open field test, although significant statistical difference was not found (Figure 5(a)). Concerning the rearing numbers, none of the treatments tested displayed any effect in the reserpine-injected mice (Figure 5(b)).

### 3.6. Repeated Administration of RSV, RO-Combined RSV Reduces the Immobility Time of the Mice in the Fibromyalgia Model

**Figure 6:**
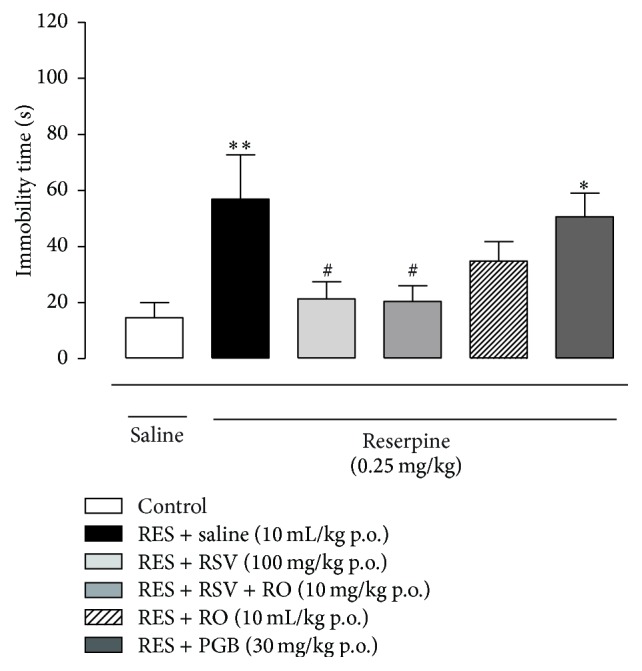
Effects of chronic treatment with RSV, RSV + RO on the forced swimming test in the fibromyalgia model in mice. On the 4th day after reserpine injection (0.25 mg/kg, s.c., once a day for 3 days), the mice treated during 4 days with SAL, RSV, RSV + RO, RO, or PGB were submitted to the forced swimming test. Immobility time was assessed during 2 min. Data are presented as mean + SEM. ^*∗*^ *p* < 0.05, ^*∗∗*^ *p* < 0.01 compared to control (SAL-SAL), ^#^ *p* < 0.05 compared to RES-SAL group. Statistical analysis was performed by one-way ANOVA followed by Bonferroni's* post hoc* test. *n* = 6 to 10 mice/group.

The repeated injection of reserpine (0.25 mg/kg once a day for three days) resulted in an increased immobility time in the forced swimming test when compared to the control group (SAL-SAL; Figure 6). Mice treated with RSV (100 mg/kg once a day for four days), RSV + RO (10 mg/kg once a day for four days) displayed a reduced immobility time in the forced swimming test in comparison to the RES-SAL group (*p* < 0.05, Figure 6). RO and PGB treatments failed to affect the immobility caused by reserpine injections (*p* > 0.05).

### 3.7. Assessment of Serum Transaminases

**Figure 7:**
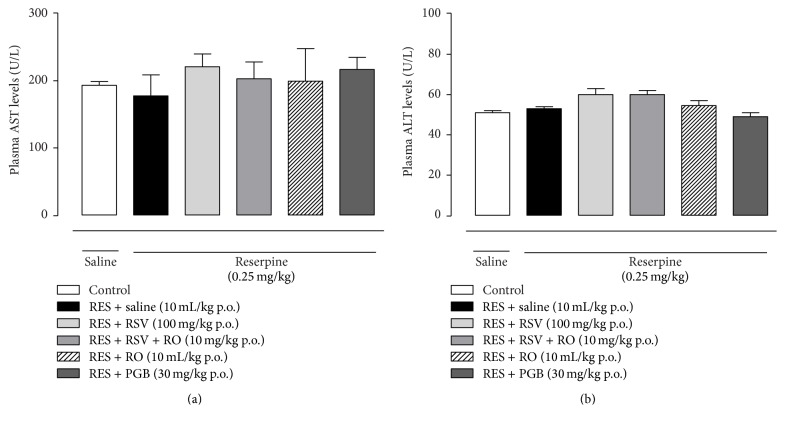
Levels of AST and ALT transaminases in the plasma of reserpine-treated mice in the fibromyalgia model. AST and ALT enzymes were measured in the plasma of reserpine-injected mice after the treatment with RSV, RSV + RO, RO, or PGB (once a day for 4 days). Data are presented as mean ± SEM. Statistical analysis was performed by one-way ANOVA. (a and b) *n* = 6 to 10 mice/group.

AST and ALT enzymes were evaluated in the serum of the mice on the 4th day after the onset of reserpine injection (0.25 mg/kg once a day for three days). The corresponding treatment administration was as follows: RSV (100 mg/kg once a day for four days), RSV + RO (10 mg/kg once a day for four days), RO (10 mL/kg once a day for four days), or PGB (30 mg/kg once a day for four days). As depicted in Figure 7, we observed that the serum levels of AST and ALT enzymes were not affected significantly by any of the protocols of treatment tested in the reserpine-induced fibromyalgia in mice (*p* > 0.05).

### 3.8. Treatment with RSV, RO-Combined RSV, RO, or PGB Diminishes RS Levels in the CSF of Reserpine-Induced Fibromyalgia Model

**Figure 8:**
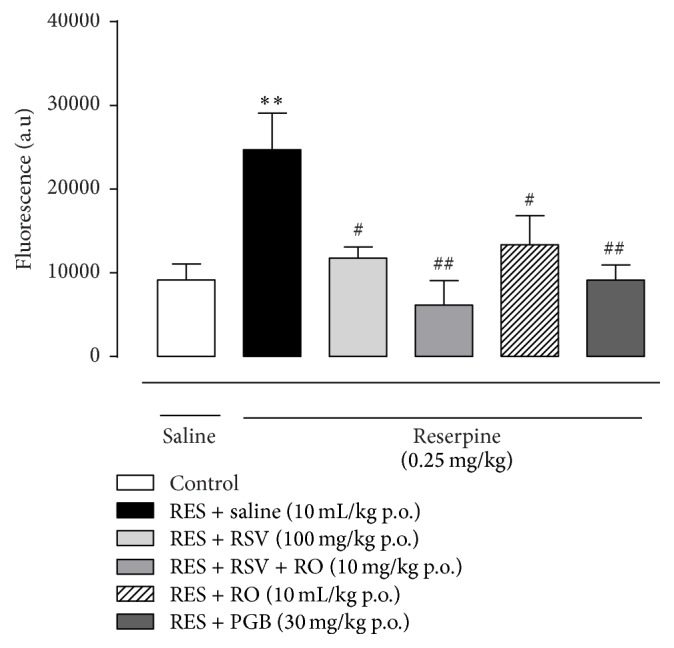
Chronic treatment with RSV, RSV + RO, RO, or PGB reduces ROS production in the CSF of reserpine-treated mice in the fibromyalgia model. ROS production was measured in the CSF of reserpine-injected mice after the treatment with RSV, RSV + RO, RO, or PGB (once a day for 4 days). Data are presented as mean + SEM. ^*∗∗*^ *p* < 0.01 compared to control (SAL-SAL), ^#^ *p* < 0.05, ^##^ *p* < 0.01 compared to RES-SAL group. Statistical analysis was performed by one-way ANOVA followed by Bonferroni's* post hoc* test. *n* = 5 mice/group.

Additionally, we aimed, to the best of our knowledge of how the (anti)nociceptive activity occurs, to measure the RS levels in the CSF of reserpine-treated mice. The repeated injection of reserpine (0.25 mg/kg once a day for three days) produced a significantly increased amount of RS in the CSF in comparison to the control group (*p* < 0.001, Figure 8). When we evaluated the changes caused by RSV (100 mg/kg once a day for four days), RSV + RO (10 mg/kg once a day for four days), RO (10 mL/kg once a day for four days), or PGB (30 mg/kg once a day for four days) treatments, we observed that RS levels in the CSF diminished in relation to the RES-SAL group (*p* < 0.05, Figure 8).

## 4. Discussion

Fibromyalgia is a complex painful disorder associated with other symptoms, leading to a multidisciplinary approach for its treatment [45]. Pharmacological management of FM is often associated with nonpharmacological approaches. The available drugs to treat FM symptoms might include several classes of analgesics, sedatives, antidepressants, and other drugs [46]. Nevertheless, not all are well tolerated [47, 48] and they do not cover the broad range of FM-related symptoms [45], thereby raising the necessity of finding new drugs. Addressing this approach, Nagakura et al. [13] validated a rat model of fibromyalgia and de Souza et al. [14] adapted the model for mice. In an attempt to find new compounds to treat FM, previously reported mice model of FM has been used. There is much evidence in the literature showing the benefits of RSV in various diseases, which led us to search for possible effects of RSV in the FM model.

Considering the involvement of impaired antioxidant defenses of the organism in the development of diseases such as cardiovascular [49], inflammatory [50], tumorigenic [51], neurodegeneration [52], and neuropathic [14] ones, similar oxidative stress processes might be involved in the pathological events underlying FM. To assess this possible mechanism, the potential role of nutraceutical antioxidant compounds RSV and RO isolated or combined in reverting behavioral changes induced in FM-like model and measuring RS levels was tested.

In this circumstance acute and chronic effects of the administration of RSV, RO, or RO-combined RSV in mice on nociceptive and depressive-like behavior in a model of FM were accessed. The acute administration of RSV produced an increase in the mechanical allodynia threshold, but not in the hot plate test compared to the administration of saline in the reserpine-injected mice. On the other hand, the analgesic effects of chronic administration of RSV displayed a different pattern: RSV increased either the mechanical allodynia or hot plate test thresholds, similar to the positive drug control PGB. These data indicate the antinociceptive role of RSV for the treatment of painful symptoms of FM. The antinociceptive effects of RSV in acute and chronic inflammatory pain models in rodents have already been reported [21, 22], suggesting a preventive analgesic role for this compound; additionally, it was also demonstrated that RSV is able to relieve diabetic neuropathic pain [53].

There is a lack of data investigating whether RO* per se* displays antinociceptive effects. In the present FM model, it has been noticed that RO is not able to produce analgesic effects, which was demonstrated through mechanical allodynia and hot plate tests. However, the chronic administration of RO itself almost reversed thermal hyperalgesia in the hot plate test (*p* = 0.0508 compared to RES-SAL group). Interestingly, Souto et al. [38] demonstrated a synergistic therapeutic effect of RSV and RO against an acute model of inflammation and a model of polyarthritis induced in rats. Then, we assessed whether the RSV transported in RO displays analgesic effects in the FM model. It has been noted that both acute and chronic schemes of administration of RO-combined RSV exhibited antinociceptive effects, demonstrated by the increased mechanical and thermal threshold levels performed through the mechanical allodynia and hot plate tests, indicating the potential therapeutic effect of the combination of RSV and RO to treat FM-painful related symptoms.

The locomotive activity of treated mice in the open field test was also assessed. It was observed that neither acute nor chronic treatment was able to reverse the locomotive behavior induced by the repeated injections of reserpine. The depressive behavior associated with FM only in the chronic scheme of administration was assessed and it was observed that the depressive behavior was prevented by the repeated administration of RSV and RO-combined RSV, returning to the control situation; this did not happen with the repeated administration of RO only. The immobility time data are in accordance with the previous data published by Souto et al. [38], in which the depressive behavior in rats induced by inflammatory states was prevented by the chronic administration of RSV and RO-combined RSV.

The results concerning the use of RSV transported in RO represent a promising therapeutic alternative for the treatment of symptoms related to several diseases, including FM, as we have presented here. The therapeutic approach of RO-combined RSV is reinforced by data published by other groups, in which the effects of the RSV were improved by lipid core nanocapsules [30, 54].

In an attempt to provide information concerning the mechanism of action for the antinociceptive effect of RSV and RO in this model, the antioxidant potential of both nutraceutical compounds was analyzed [38]. For this purpose, the RS levels in the CSF of the animals have been assessed, using the DCFH assay. Interestingly, when dosed daily after reserpine injections, all treatments prevented the rise in the RS levels present in the reserpine-injected mice receiving only saline solution. These data reinforce the antioxidant ability of the RSV, corroborating a wide range of works which show the beneficial antioxidant effects of RSV in cardiovascular [23] and inflammatory disease [21], hepatic steatosis [29], cancer [31], diabetic neuropathy [55], and antiageing alterations [56] and the antioxidant ability of RO as hypolipidemic agent [36], anti-inflammatory [35], and others.

The involvement of oxidative stress has been related to the symptom of fatigue [57], a coexistent symptom in addition to widespread pain in FM patients. Some researchers investigated the role of oxidative stress in the pathological processes underlying FM. Akbas et al. [58] have observed elevated superoxide dismutase (SOD) antioxidant enzyme activity in patients with FM compared to healthy control patients, suggesting an increased oxidative stress. Furthermore, data has been published showing low total antioxidant status associated with high total oxidant status and oxidative stress index in patients with FM according to control groups [59]. Remarkably, mitochondrial dysfunction was demonstrated to be present in patients with FM, through the expression of transcription factor A mitochondrial (TFAM) and peroxisome proliferator-activated receptor gamma-coactivator 1-alpha (PGC-1*α*), the mitochondrial factors involved in mitochondrial biogenesis [60]. Although we have not investigated mitochondrial biogenesis, we can deduce that mitochondrial pathways are involved in the present fibromyalgia-like model, since the mitochondria is responsible for RS production, and changes were observed in this parameter with the increased RS levels in reserpinized mice prevented by the antioxidant compounds RSV and RO.

Moreover, the CSF increased levels of RS present in reserpine-injected mice observed here are in accordance with the results noted by other researchers, in which the RS production is involved in persistent pain arising from injury [61] or inflammatory insult [62]. Based on the ability of the association of RSV and RO in preventing increased RS levels observed here, focusing on antioxidant compounds can be an alternative for holding the oxidative stress. Thus, we might assign a relationship between RS levels and nociceptive behavior in reserpine-injected mice. Although further investigation should be made about the underlying pathophysiological mechanism of FM, it was noted that the association of RSV and RO might be a therapeutic option for FM, since they present preventive antinociceptive and antioxidant actions on the fibromyalgia-like model.

The animal model used in this study is based on the ability of reserpine in depleting biogenic amine (serotonin, glutamate, and dopamine) in the central nervous system [13]. A question which has not been explored yet concerning reserpine is the impact of the repeated administration of this drug for liver functional parameters. In order to answer this question, the serum levels of ALT and AST obtained from mice were assessed. ALT and AST are transaminases enzymes, with increment in serum levels that indicate hepatic lesion [63]. Results showed no alteration in serum transaminases levels of reserpine-injected mice, with similar values to those observed in control group. This data suggests that the reserpine injections cause no change in liver function according to biochemical activity, which could be noted at least in a subchronic scheme of administration.

Strikingly, data presented here show that treatment with RSV transported in RO (10 mg/kg +10 mL/kg), in a dose 10-fold lower than treatment with RSV only (100 mg/kg), reversed nociceptive thresholds back to control levels and had higher thresholds than the RSV only group. Based on this, we suggest at least an additive antinociceptive effect of combining RSV and RO. This effect may be due to delayed delivery of RSV into the system when it is in an oil-blend composition. The effects of RSV and RO in the fibromyalgia-like model may be due to diminishment of oxidative stress and reduced RS levels in CSF in the mice. RS has been implicated in the development of persistent pain resulting from injury or inflammatory insult [62]. Agents reducing RS have been demonstrated displaying antihyperalgesic action [61]. Bazzo et al. [21] suggest that RSV might be a viable alternative in pain management through its powerful antioxidant activity. Antioxidant compounds, such as polyphenols, tocopherols, and tocotrienols, have been proposed to have beneficial effects on human health by preventing cellular damage and the development of chronic diseases [27, 34]. Additionally, recent data shows that the serotoninergic system contributed to the antinociceptive and antidepressant action of RSV in a mouse model of neuropathic pain [64]. It is possible that both of the above-mentioned mechanisms are involved in the beneficial activity of RSV on fibromyalgia pathology.

In conclusion, we demonstrated herein a novel possible mechanism involved in the model of reserpine-induced fibromyalgia additional to the depletion of biogenic amine, despite further studies being needed. Reserpine was not shown to alter the hepatic function. Moreover, the potential of analgesic action of the RSV and RO association in treating fibromyalgia-related symptoms in a mouse model was also demonstrated. Because of the broad range of applicability of RSV and RO as a nutraceutical product and its relevant antioxidant and antinociceptive activities, the approach presented by us can be easily applicable in rheumatologic clinics as a pharmacological option for the treatment of FM.
